# Consequences of the Timing of Menarche on Female Adolescent Sleep Phase Preference

**DOI:** 10.1371/journal.pone.0005217

**Published:** 2009-04-22

**Authors:** Sylvia Frey, Silvia Balu, Sarah Greusing, Nicolas Rothen, Christian Cajochen

**Affiliations:** 1 Centre for Chronobiology, Psychiatric University Hospital, University of Basel, Basel, Switzerland; 2 Institute of Psychology, University of Bern, Bern, Switzerland; Vanderbilt University, United States of America

## Abstract

Most parents experience their children's puberty as a dramatic change in family life. This is not surprising considering the dynamics of physical and psychosocial maturation which occur during adolescence. A reasonable question, particularly from the parents' perspective, is: when does this vibrant episode end and adulthood finally start? The aim of the present study was to assess the relationship between puberty and the changes in sleep phase preferences during female maturation and adulthood by a cross-sectional survey. The results from 1'187 females aged 5 to 51 years based on self-report measures of sleep preferences on weekdays and on free days as well as the occurrence of menarche, show that in contrast to prepubertal children, adolescent females exhibit a striking progression in delaying their sleep phase preference until 5 years after menarche. Thereafter, the sleep phase preference switches to advancing. The current study provides evidence that a clear shift in sleep-wake cycles temporally linked to menarche heralds the beginning of “adult-like” sleep-wake behaviour in women and can be used as a (chrono)biological marker for the onset of adulthood.

## Introduction

Most parents experience their children's puberty as a dramatic change in family life. This is not surprising, considering the dynamics of physical and psychosocial maturation, which occur during adolescence. A reasonable question, also from the parents' perspective, is: when does this vibrant episode end and adulthood finally start?

Human sleep-wake behaviour undergoes a 24-h rhythm, which is governed by the biological clock located in the suprachiasmatic nuclei [1]. However, individual sleep and wake time preferences are fairly diverse due to genetic, environmental and age-related factors, resulting in different individual timing (phase position) for early chronotypes (larks) and late chronotypes (owls) [2]–[8].

One striking characteristic of adolescence is a marked delay of sleep phase preference and an enhanced sleep duration on weekends that contrasts with sleep timing during weekdays [9]–[11]. As wake times during weekdays are fairly constant because of school times, and only bedtimes shift later, a considerable sleep deficit accumulates prior to the weekend. This deficit has to be caught up over the weekend, and is attempted by a temporal shift of wake and sleep times. The difference in sleep timing preference between weekdays and weekends has been aptly described as “social jetlag” [12].

Apart from the more obvious psychosocial factors, delayed phase preference in adolescents may actually be related to changes in the phase of the circadian timing system. A later pubertal development stage coincides with a later chronotype, particularly in females [13], [14].

In contrast to the end of the biological maturation (puberty), which is associated with a stop in physical growth, the end of other maturation domains of adolescence such as developing cognitive skills (e.g. formal operational thoughts) and psychosocial competence (e.g. identity formation) is yet not well defined. From this perspective, the beginning of adulthood still remains a fuzzy area. Recently, Roenneberg et al. [15] have suggested that the age- and sex-dependent switch from delaying to advancing chronotype may represent a biological marker for the end of adolescence.

However, chronological age alone does not give an accurate explanation for the physical and behavioural dynamics that occur before the transition from adolescence to adulthood. Thus, it remains unclear whether there is a relation between puberty and this switch in sleep timing preference at the end of adolescence. Here we aimed at assessing a relationship between puberty and the changes in sleep phase preferences during female maturation and adulthood.

## Methods

Our study is based on a survey among 1'187 females aged 5 to 51 years (mean 23.7 years, SD = 9.5 years; median = 20 years; see supporting Fig. S1) of whom 2% were nursery girls, 40.5% high school students, 16.2% university students, and 41.3% of unknown occupation. 44.7% of the study participants were surveyed during the summer months (June–August), 33.6% during winter (December–February), 11.9% during spring (March–May), and 9.8% during fall (September–November).

Study participants were mainly recruited at high schools and at the University of Basle, Switzerland. Schoolmasters and teachers as well as University tutors were asked for approval and support of the survey. In addition to the survey on paper used at high schools and at the University an electronic version of the questionnaire was freely available online on our website (www.chronobiology.ch). Approximately 80% of the questionnaires were distributed personally by our researchers, teachers, and University tutors whereas about 20% were filled out anonymously online. Apart from the latter, study participation was either with verbal or written consent from the parents (nursery children). The study procedure and questionnaire were approved by the local Ethics Committee of Basle (EKBB), Switzerland, and all procedures conformed to the Declaration of Helsinki.

**Figure 1:**
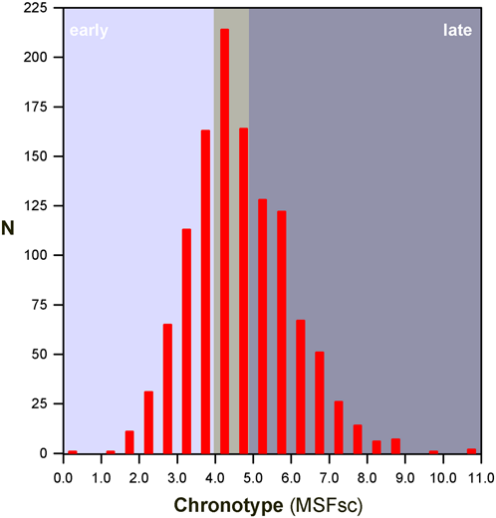
Chronotype distribution within the study sample. The MSF_sc score reflects the midpoint of sleep and therefore time of day (N = 1187).

**Figure 2:**
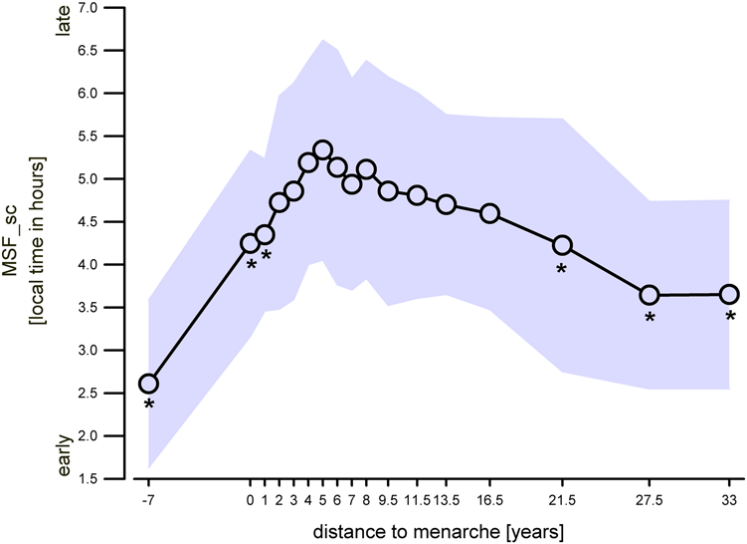
Development of sleep phase preference with reference to pubertal maturation. Chronotype as indexed by the MSF_sc value is a measure for sleep phase preference. MSF_sc represents local time in hours. Shaded area represents±SD; * indicate significant values compared to 5 years after menarche (Bonferroni adjusted alpha levels, for further statistics please see text).

**Figure 3:**
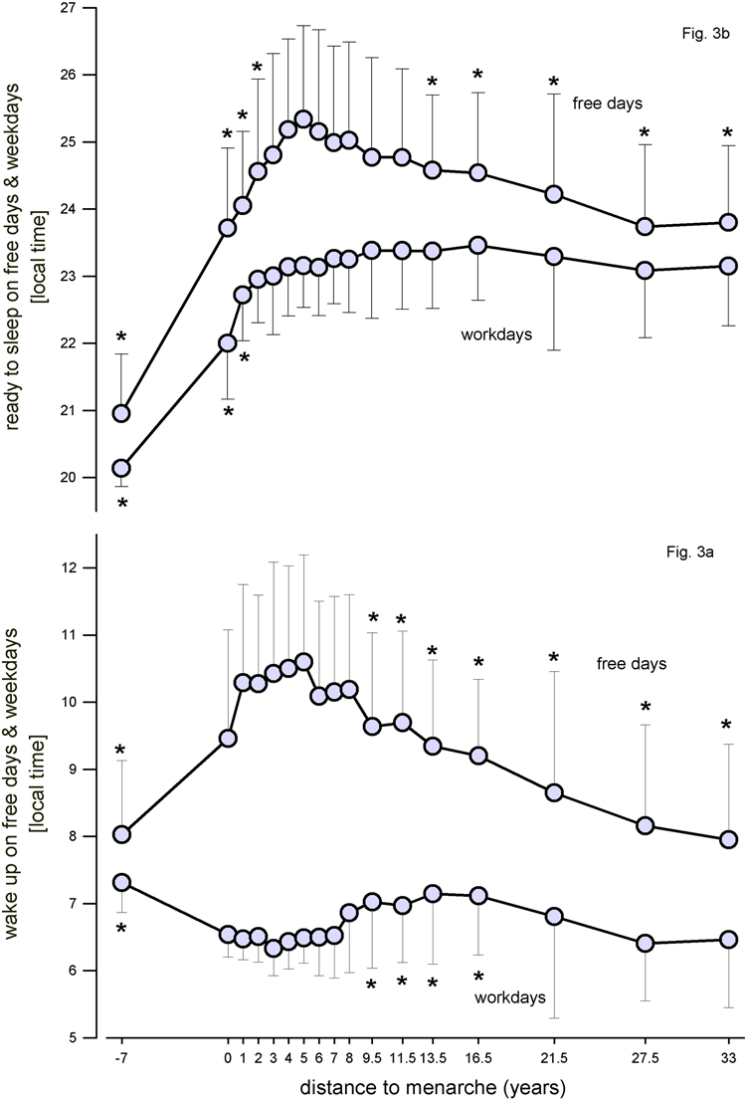
Sleep and wake up times during free days and weekdays with reference to pubertal maturation. *Mean values*; *N* = *1187*±*SD*; * indicate significant values compared to 5 years after menarche (Bonferroni adjusted alpha levels, for further statistics please see text).

Chronotypes were assessed by the Munich Chronotype Questionnaire (MCTQ) [16]. The chronotype score “MSF_sc” was used to analyse differences in sleep phase preferences (Fig. 1 and Fig. 2). MSF_sc is calculated by an adjustment of the midpoint of sleep on free days by the individual average sleep need throughout the week as described in the supplemental data to [15]. Furthermore, habitual sleep timing on weekdays and free days were investigated (Fig. 3).

Along with the date of birth, the year and month of menarche occurrence was also requested. Menarche was chosen as a pubertal marker since it is a milestone which remains mentally present throughout a woman's life. The distance measured in years from the present age to menarche was taken as a biological variable that may affect the change in sleep timing preference. Being age-dependent, this variable may serve as an indirect measure of the stage of pubertal maturation.

Although menarche timing was assigned by retrospective self-reported data, the observed mean age at menarche in our study of 12.96 years+/−1.41 years (median = 13 years; n = 1140) matched longitudinally measured age of menarche in Western societies ranging from 12.6 to 13.4 years rather well [17]–[20]. Importantly, several studies have reported a moderate to high correlation between the real age at menarche and the menarche age recalled during adulthood and adolescence [21]–[25]. In order to avoid bias with reference to the exact month of occurrence of menarche, only the year of menarche was considered in the present analysis.

**Figure 4:**
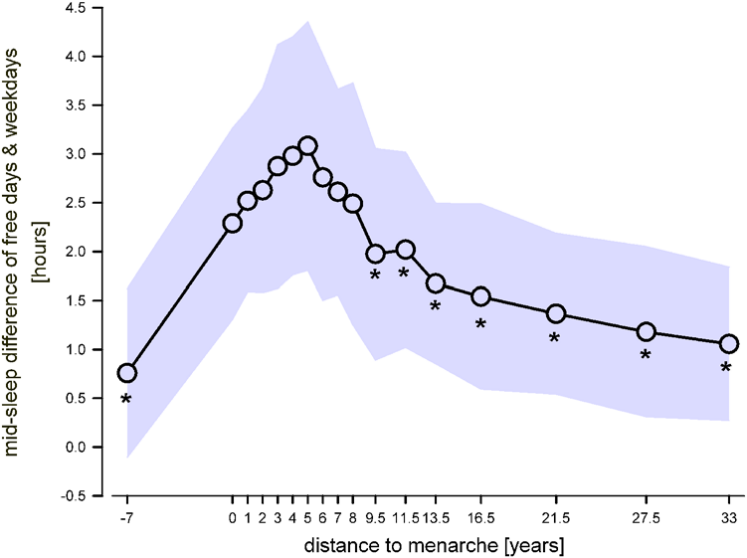
Difference in sleep midpoints between free days and weekdays with reference to pubertal maturation. Mid-sleep represents the average difference between sleep phase midpoints on free days and sleep phase midpoints on weekdays. A striking switch-over from a consecutive delaying of the sleep midpoint difference to an advancing occurs 5 years after menarche. Shaded area represents±SD; * indicate significant values compared to 5 years after menarche (Bonferroni adjusted alpha levels, for further statistics please see text).

**Figure 5:**
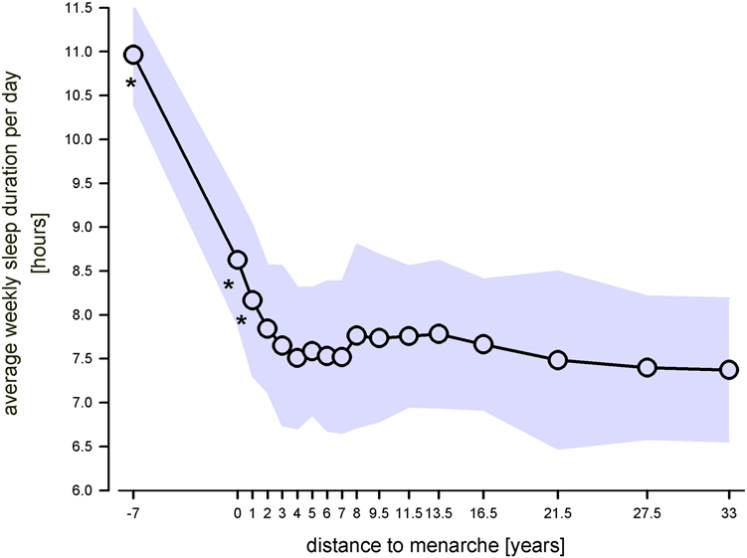
Average weekly sleep duration per day with reference to pubertal maturation. Average weekly sleep duration per day reflects the average value calculated on the basis of 5 weekdays and 2 free days. Two years after menarche the average sleep duration per day remains remarkably stable between 7.5 and 8 hours for the remainder ‘distance to menarche’ classes. Shaded area represents±SD; * indicate significant values compared to 5 years after menarche (Bonferroni adjusted alpha levels, for further statistics please see text).

**Figure 6:**
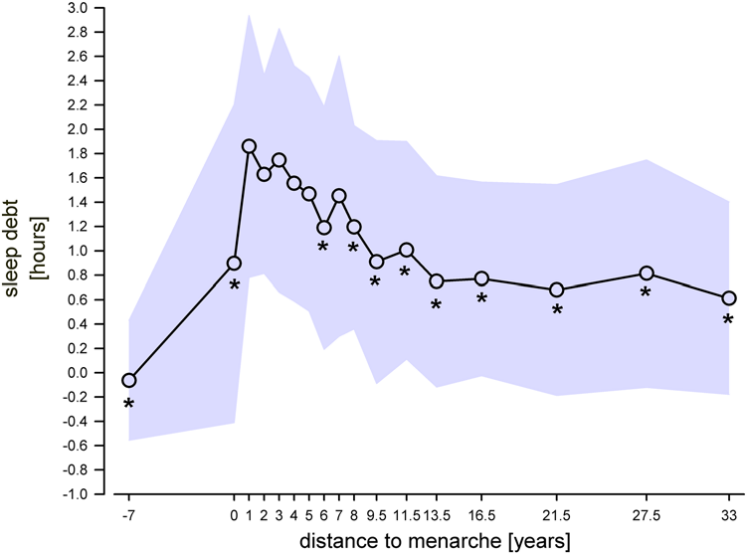
Sleep debt with reference to pubertal maturation. Sleep debt represents the amount of sleep which is compensated for during free days due to a chronic sleep loss due to social demands during weekdays. The highest level of sleep debt occurs 1 year after menarche. Afterwards, a decrease in sleep debt was observed. Shaded area represents±SD. * indicate significant values compared to 1 year after menarche (Bonferroni adjusted alpha levels, for further statistics please see text).

To account for interferences of individual sleep preference with social demands the difference between the midpoints of sleep on free days and on weekdays was calculated (Fig. 4). The assessment of mid-sleep time was calculated on the basis of indicated sleep onset and wake up times in the questionnaire. Average sleep duration (Fig. 5) was calculated by the formula (5*sleep duration on weekdays+2*sleep duration during free days) / 7) according to the supplemental data to [15]. Sleep debt accumulated during the weekdays which is compensated on free days is calculated according to the supplemental data to [15] and illustrated with reference to distance to menarche (Fig. 6).

Distance to menarche as shown in Figures 2, 3, 4, 5, and 6 was calculated as the difference between actual age and age at menarche. “0” corresponds to survey participants who did not yet experience menarche but are considered to be “close” to it because of their actual age ranging from 10–17 years (mean = 12.83 years, SD = 1.53 years; median = 13 years; n = 23). The difference between the median age of this group and the median age at menarche in our sample is 0 and therefore the distance to menarche within the graph was considered accordingly. A distance to menarche of −7 years corresponds to prepubertal girls (mean age = 5.7 years, SD = 0.49 years; median = 6 years; n = 24). The distribution of the sample into ‘distance to menarche’ classes is displayed in supporting Figure S2.

The variables ‘midpoint of sleep difference’ and ‘MSF_sc’ were log-transformed in order to achieve a normal distribution of the data set (normal distribution confirmation by Kolmogorov-Smirnov test). Log-transformed values of these two variables were afterwards subjected to a one-way ANOVA each with the factor ‘distance to menarche”. Post-hoc comparisons were based on the LSMEANS procedure in SAS with a Bonferroni alpha level correction. Statistical analysis on the variables concerning bedtimes and wake up times, sleep duration as well as sleep debt were based on the Kruskal Wallis test. Post-hoc comparisons were based on the Mann-Whitney U Test with a Bonferroni corrected alpha level. Statistical analyses were performed with the statistical packages SAS (version 9.1) and Statistica (version 6.1).

## Results

Figure 1 displays the distribution of chronotypes in our sample. About 36.4% of the survey participants were late chronotypes whereas early and intermediate chronotypes accounted for 32.3% and 31.3%, respectively. An examination of chronotype distribution with respect to pubertal maturation (i.e. ‘distance to menarche’) confirmed previous findings that evening types are more prevalent during adolescence and morning types in children as well as in females above 30 years (supporting Fig. S3).

As expected, prepubertal children exhibited a relatively early sleep phase preference compared to females during developmental maturation (Fig. 2). A peak value in sleep phase preference occurred 5 years after menarche and was therefore chosen in the following as reference point for post-hoc analyses unless mentioned otherwise. After this time point chronotype advanced progressively with years since menarche. One-way ANOVA yielded significance for the factor ‘distance to menarche’ (p<0.0001). Post-hoc comparison tests yielded that MSF_sc at −7 years to menarche (p<0.0001), close to menarche (p<0.05), 1 year after menarche (p<0.001), 21.5 years, and more after menarche (p<0.0001) were significantly different from the value at 5 years after menarche. Additionally, the difference of the MSF_sc at 16.5 years after menarche to the peak at 5 years after menarche was found to be close to significance with p = 0.0505.

Habitual sleep and wake up timing on free days and weekdays are shown in Figures 3a and 3b. Kruskal Wallis test yielded significance for each of the variables for the factor ‘distance to menarche’ (p<0.001). Post-hoc comparisons of bedtimes during the weekdays showed no significant difference between 2 years and 33 years after menarche. Taking the reference point (5 years after menarche) into account, only the bedtimes before the occurrence of menarche and 1 year after menarche differed significantly (p<0.003). In contrast, wake up times during weekdays 5 years after menarche differed significantly from several wake up times at earlier and later maturation periods (−7, 9.5, 11.5, 13.5, and 16.5 years after menarche; p<0.003). Bedtimes during free days revealed significant differences of the ‘distance to menarche’ classes at −7, 0, 1, 2, 13.5, 16.5, 21.5, 27.5, and 33 years compared to 5 years after menarche (p<0.003). Regarding the wake up-times during free days there were significant differences at −7 years to menarche and from 9.5 to 33 years after menarche compared to 5 years after menarche (p<0.003).

Prepubertal children exhibited a small shift in sleep timing preference between free days and weekdays (Fig. 4). As shown in Fig. 2, a delayed sleep phase preference was observed after the occurrence of menarche, which progressively increased until 5 years after menarche where the mid-sleep difference between weekdays and free days reached a maximum of about 3 hours on average (Fig. 4). Afterwards, a progressive decrease of the mid-sleep difference was observed. One-way ANOVA yielded significance for the factor ‘distance to menarche’ (p<0.0001). Post-hoc comparison showed that mid-sleep difference at −7 years to menarche and from 9.5 years and more after menarche were significantly shorter as the peak in mid-sleep difference at 5 years after menarche (p<0.0001).

The average weekly sleep duration per day decreased remarkably from about 11 hours at prepuberty to less than 8 hours 2 years after menarche (Fig. 5). With progressive maturation (two years and more after menarche) average sleep duration levelled off between 7.5 and 8 hours. A Kruskal Wallis test yielded significance for the factor ‘distance to menarche’ (p<0.001). Post-hoc comparisons showed that only the average weekly sleep duration per day before the occurrence of menarche and 1 year after menarche was significantly different from the reference value at 5 years after menarche (p<0.003). Furthermore, post-hoc comparisons revealed that after 2 years after menarche only values at 27.5 years and 33 years after menarche differed significantly from the value at 2 years after menarche (p<0.003).

The highest sleep debt level occurred 1 year after menarche and amounted to almost 2 hours (Fig. 6). According to the Kruskal Wallis test the time course of the sleep debt with respect to distance to menarche was significant (p<0.001). Post-hoc comparisons confirmed that sleep debt at 1 year after menarche was significantly different from the sleep debt before menarche and also from 6, 8 and more years after menarche (p<0.003).

## Discussion

Changing psychosocial pressures during adolescence, such as increased evening leisure activities and earlier morning school schedules are important influences on sleep timing. Until now, the biological underpinnings of physiological sleep-wake cycles during maturation have been neglected. These regulatory processes seem to undergo realignment in phase relationships which may favour delayed sleep patterns in adolescents [14].

Our data, which show a distribution of chronotypes similar to an extensive sample mainly from Germany, Switzerland, and Austria illustrated in [12], [15], clearly point towards a temporal association between a biological marker of puberty and sleep phase preference during and after maturation. Moreover, our data provide evidence that the increased interference between individual sleep preference and social demands - as indexed by the difference of sleep midpoints on free and week-days - cannot be explained solely by an accumulation of a sleep debt after menarche lasting until 5 years thereafter. Thus, the difference in sleep duration between week and free days (or sleep debt as shown in Fig. 6) is relatively low before menarche, highest just after experiencing menarche, and decreases while the sleep phase preference is delaying until 5 years after menarche. However, on the basis of our data, we cannot fully differentiate between social and biological influences on the evolution of sleep phase preference during maturation.

Studies have shown that as a consequence of social jetlag accumulated during the week (which increases on average up to 3 hours as shown here), there is an increased risk for mood dysregulation, impaired school performance, excessive daytime sleepiness, and addictive drug abuse in adolescents [12], [14], [26]–[30]. Furthermore, Knutson [31] reports that particularly females show a significant correlation between pubertal development and an increased risk of insomnia and waking tired. So far, chronobiological interventions to the circadian timing system, such as exposure of adolescents to bright light in the morning or scheduled naps, did not show any stabilising effect on the sleep-wake cycle or on performance [32], [33].

One important question about the underlying processes responsible for the switch to advance in sleep timing preference which occurs after 5 years after menarche remains to be examined. Is there a change in nocturnal plasma melatonin levels at this developmental stage other than the observed pubertal decline of this hormone [34]–[37]? It has been suggested that body size acts as mediator for the plasma melatonin decline during puberty [38]. Hence, does attainment of adult body size mediate a change in the circadian signal, which leads to the alteration in circadian phase preference? Our results provide evidence for a specific maturation-dependent time point to be considered in such in-depth analysis.

We conclude that the downsizing of social jetlag 5 years after menarche is a step towards “adult-like” behaviour as the ability to approach more aligned sleep-wake cycles during weekdays and weekends emerges. The distance to menarche may serve as an individual biological marker for the beginning of female adulthood. Thus, to answer the parents' question: the vibrant episode of adolescence should be over 5 years after menarche.

## Supporting Information

Figure S1 Age distribution within the study sample.
(0.15 MB TIF)

Figure S2 Sample distribution with respect to distance to menarche. ‘Distance to menarche’ represents the difference between actual age and the age at the occurrence of menarche. It serves as indirect measure for pubertal maturation. A distance of −7 years to menarche means that the females in this group did not yet experience menarche. Furthermore, it means that the temporal distance to this event is 7 years based on the actual mean age of the subjects of this group compared with the median age at occurrence of menarche calculated from the sample.
(0.38 MB TIF)

Figure S3 Chronotype distribution with reference to pubertal maturation. The contour plot represents the distribution of the different chronotypes occurring at each considered time point of pubertal maturation (i.e. distance to menarche). Different colours between the contour curves indicate the magnitude of the percent portion of the chronotypes at a specific time point of pubertal maturation as indexed beside the figure. Chronotype abbreviations: E = evening type, I = intermediate type, M = morning type.
(2.82 MB TIF)
